# Controlled human exposures to ambient pollutant particles in susceptible populations

**DOI:** 10.1186/1476-069X-8-33

**Published:** 2009-07-25

**Authors:** Yuh-Chin T Huang, Andrew J Ghio

**Affiliations:** 1 Department of Medicine, Duke University Medical Center, Durham, North Carolina, USA; 2 Human Studies Division, National Health Effects Research Laboratory, US Environmental Protection Agency, Chapel Hill, North Carolina, USA

## Abstract

Epidemiologic studies have established an association between exposures to air pollution particles and human mortality and morbidity at concentrations of particles currently found in major metropolitan areas. The adverse effects of pollution particles are most prominent in susceptible subjects, including the elderly and patients with cardiopulmonary diseases. Controlled human exposure studies have been used to confirm the causal relationship between pollution particle exposure and adverse health effects. Earlier studies enrolled mostly young healthy subjects and have largely confirmed the capability of particles to cause adverse health effects shown in epidemiological studies. In the last few years, more studies involving susceptible populations have been published. These recent studies in susceptible populations, however, have shown that the adverse responses to particles appear diminished in these susceptible subjects compared to those in healthy subjects. The present paper reviewed and compared control human exposure studies to particles and sought to explain the "unexpected" response to particle exposure in these susceptible populations and make recommendations for future studies. We found that the causes for the discrepant results are likely multifactorial. Factors such as medications, the disease itself, genetic susceptibility, subject selection bias that is intrinsic to many controlled exposure studies and nonspecificity of study endpoints may explain part of the results. Future controlled exposure studies should select endpoints that are more closely related to the pathogenesis of the disease and reflect the severity of particle-induced health effects in the specific populations under investigation. Future studies should also attempt to control for medications and genetic susceptibility. Using a different study design, such as exposing subjects to filtered air and ambient levels of particles, and assessing the improvement in biological endpoints during filtered air exposure, may allow the inclusion of higher risk patients who are likely the main contributors to the increased particle-induced health effects in epidemiological studies.

## Introduction

Epidemiologic studies have established an association between exposures to air pollution particles and human mortality and morbidity at concentrations of particles currently found in major metropolitan areas [1]. This association has been documented in numerous investigations around the world and is remarkably consistent [1-8]. The adverse effects of particulate matter (PM) include both pulmonary and extrapulmonary morbidity and mortality. It is estimated that the daily cardiopulmonary mortality increased by 0.3% for each 10-μg/m^3 ^increase in PM_10 _(particulate matter < 10 μm in aerodynamic diameter). For long term cardiopulmonary mortality, the estimate was 6% for each 10-μg/m^3 ^increase in annual average exposure to PM_2.5 _(PM < 2.5 μm) [5]. The risk is especially high in the elderly and patients with chronic obstructive lung disease, asthma, coronary artery disease, congestive heart failure and frequent arrhythmias [9-14]. The adverse pulmonary effects after PM exposure include increased hospital admissions, pulmonary infections, asthma attacks, and exacerbations of chronic obstructive pulmonary disease [10,15]. The extrapulmonary adverse effects of PM are primarily cardiac diseases [1,5,16,17] and vascular diseases (e.g., ischemic stroke) [18-21].

Controlled human exposures have been used to support the association between PM exposure and adverse health effects observed in epidemiological studies. Earlier controlled human exposure studies invariably enrolled young healthy subjects. These studies largely confirmed the capability for PM to produce adverse health effects shown in epidemiological studies. In the last few years, more studies involving susceptible populations (e.g., elderly and patients with coronary artery disease, chronic obstructive lung disease and asthma) have been published [22-27]. It is somewhat surprising that many studies showed that these susceptible populations seem less responsive to PM exposure compared to healthy individuals. This paper will review all controlled exposure studies to PM in patients with cardiopulmonary diseases and compare the health effects of PM observed in these susceptible populations with those in healthy subjects in human controlled in exposure studies in an attempt to explain the seemingly "paradoxical" response to PM in the susceptible populations. We will discuss factors that may affect the PM endpoints and provide recommendations for future studies involving susceptible populations. Exposure studies that employed only PM components, e.g., endotoxins or co-pollutant gases, e.g. SO_2_, NO_2 _and CO, are beyond the scope of this review and will not be included.

### Controlled exposures to ambient pollutant particles

Controlled human exposure studies refer to those that place study subjects in an exposure chamber into which exogenous particles are introduced. Over the years, the controlled human exposure studies have greatly enhanced our understanding in PM-induced health effects, especially in apportioning the specific effects of particle constituents and the mechanisms of the adverse effects. There are, however, important limitations of controlled human exposure studies.

Relative to animal and in vitro exposure studies, controlled human exposures employ particles at concentrations more relevant to the ambient level, but there are limitations. Unlike animal and in vitro exposure studies, the particle concentration to which the subject is exposed is usually kept relative low for safety reasons. The duration of the one-time exposure is usually short (1-2 hours). As such, the biological effects may be small and the signal-noise ratio can be unfavorable. Intermittent mild-to- moderate exercise on a cycle ergometer is usually incorporated in the controlled exposure studies. Although this can increase the dose of exposure, the interpretation of some endpoints, e.g., HRV and DLCO, may be complicated by the physiological effects induced by exercise itself. The short one-time exposure makes it particularly difficult to relate a negative result to "real world" exposure, which most commonly is prolonged and repetitive. In addition, the study endpoints for controlled human exposures are limited to samples that are easily accessible, e.g., pulmonary function, blood, sputum, ECG (for heart rate variability) and sometimes bronchoscopy specimens (bronchoalveolar lavage fluid, bronchial brushing and biopsy). It may be difficult to extrapolate changes in these physiological and biochemical parameters to the endpoints frequently employed in epidemiological studies, such as mortality, hospital admission and emergency room visits.

Controlled human exposures may employ particles collected on filters (and later given by nebulization), emission and crustal source particles, surrogate particles (e.g., carbon black) or ambient air pollution particles (with or without concentration). The recovery of particles on filters can be variable, and following such collection it is questionable whether all components can be retrieved in quantities reflecting the original particles (e.g., organics and ammonium are too volatile to be collected). In addition, the particles may be contaminated by fragments of the filter when retrieved. Processed emission and crustal source particles (e.g. residual oil fly ash, particles from National Institute of Standards and Technology or NIST) and artificially generated particles (e.g. carbon black), while contributing to and included in ambient air pollution PM, are frequently deemed not truly representative of such particles, and their use has equivalent or even greater restrictions. The direct exposure of humans to ambient air pollution (e.g. road tunnel air) has the advantage of using relatively "fresh" particles, but it may be complicated by factors that may not be easily controlled (e.g., fluctuating levels of O_3_, SO_2_, and nitrogen oxides). Even in those studies that artificially adjust the concentrations of these co-pollutant gases to maintain a constant level of these gases in the exposure chamber remained constant, interactions between particles and co-pollutants cannot be excluded. Ambient pollutant particles can also be concentrated before exposure of subjects [28]. Concentrated ambient particles (CAPS) can be used to investigate the specific effects of particles. CAPS are devoid of gaseous co-pollutants and possible interactions between PM and these co-pollutants cannot be examined unless the latter are re-introduced. CAPs exposures can also allow the investigation of specific effects of particles within a size range (e.g. PM_2.5_) by the use of a cascade impactor. Most protocols include intermittent exercise on a stationary bike during the exposure to elevate minute ventilation and thus increase the dose of particles inhaled. Volunteers are exposed sequentially or at random to filtered air and pollutant(s) of interest, although the actual protocol used varies in duration and timing. These variations in particle sources and protocols can make it difficult to directly compare results between controlled human exposure studies.

### Controlled exposures to ambient pollutant particles in patients with ischemic heart disease

Controlled exposure studies involving patients with cardiovascular diseases that are discussed in this review are summarized in Table 1. In a placebo controlled, double-blinded, randomized, crossover study, Routledge et al. [25] examined the effects of ultrafine carbon particles (at 50 μg/m^3^) as well as SO_2 _(200 ppb alone and in combination) on heart rate variability (HRV) and circulating markers of inflammation and coagulation. The control exposure was bottled medical air. Twenty patients with stable angina, multivessel coronary artery disease, and good left ventricular function (with an ejection fraction of 40%) were recruited from the waiting list for coronary artery bypass grafting. Medications were unchanged through the study period. Twenty healthy volunteers of similar ages served as the control. Subjects rested for 30 minutes before the baseline blood sample was drawn. After an additional 15 minutes, two five-minute segments of ECG, respiration, and arterial blood pressure data were recorded during controlled respiration. Subjects were then exposed to the pollutant for one hour. Blood samples and two five-minute recordings of the HRV data were obtained immediately after exposure and again at four hours. Another blood sample was taken at 24 hours. Studies of the remaining pollutants were repeated in random order seven to 14 days apart with identical protocols. In healthy subjects, exposure to ultrafine carbon particles resulted in immediate small increases in RR interval, SDNN, and rMSSD compared to air alone. Low frequency (LF) power also increased. Although high frequency (HF) power rose similarly, this did not reach statistical significance. Changes in HRV measures were no longer significantly different from those after air exposures four hours after exposure. HRV did not change significantly immediately after SO_2 _exposure, but RR interval, SDNN, rMSSD, and PNN50 were significantly reduced at four hours post-exposure, indicating a reduction in cardiac vagal control. HF power also tended to decrease, but this did not reach statistical significance. When exposures to carbon and SO_2 _were entered as factors in a repeated measure analysis of variance, there was significant interaction between carbon and SO_2_, such that the effect of SO_2 _on HRV appeared to be modulated by the presence of carbon (p < 0.001 for rMSSD and p < 0.005 for SDNN). Patients with stable angina did not show any of the above changes in HRV. There were no changes in the markers of hemostatic system, including platelet aggregation, fibrinogen and D-dimers in either healthy volunteers or patients with angina.

**Table 1:** Controlled human exposure studies in patients with ischemic heart disease and metabolic syndrome.

Study	Subject (number)	Design	Particles	Results
Routledge et al [25]	Healthy volunteers (20) Patients with multivessel coronary disease (20)	Placebo controlled, double-blind, randomized	Filtered air Carbon (50 μg/m^3^) SO_2 _(200 ppb) Carbon + SO_2_	Small increases in RR interval, SDNN, rMSSD LF power immediately post-carbon exposure; Decreased RR interval, SDNN, rMSSD, and PNN50 at 4 hours post-SO_2 _exposure

Mills et al [29]	Healthy volunteers (30)	Double-blind, randomized, crossover	Filtered air Diesel exhaust (300 μg/m^3^)	Attenuated forearm blood flow increase induced by bradykinin, acetylcholine, and nitroprusside infusion at 2 and 6 hours after exposure; Suppressed the bradykinin-induced release in plasma t-PA 6 hours after exposure

Mills et al [24]	Patients with prior myocardial infarction (20)	Double-blind, randomized, crossover	Filtered air Diesel exhaust (300 μg/m^3^)	Greater increase in the exercise-induced ischemic burden and decrement of ST segment during exposure; No effects on preexisting vasomotor dysfunction at 6 hours post-exposure; Reduced the bradykinin-induced release of endothelial t-PA

Tornqvist et al [30]	Healthy volunteers (15)	Double-blind, randomized, crossover	Filtered air Diesel exhaust (300 μg/m^3^)	Decreased endothelium-dependent vasodilatation; No effects on endothelium-independent vasodilatation or the bradykinin-induced release of endothelial t-PA

Mills et al [31]	Age-matched healthy volunteer (12) Patients with stable coronary artery disease (12)	Double-blind, randomized, crossover	Filtered air Fine and ultrafine CAPS (190 μg/m^3^)	CAPS did not affect vasomotor or fibrinolytic function in either middle-aged healthy volunteers or patients with coronary heart disease.

Peretz et al [35]	Healthy subjects (10) Metabolic syndrome (17)	Double-blind, randomized, crossover	Filtered air Diesel exhaust (100 and 200 μg/m^3^)	Decreased brachial artery diameter, increased plasma ET-1; No differences between healthy subjects and patients with metabolic syndrome

Carlsten et al [36]	Metabolic syndrome (16)	Double-blind, randomized, crossover	Filtered air Diesel exhaust (100 and 200 μg/m^3^)	Decreased vWF at 7 hours postexposure

Carlsten et al [37]	Healthy subjects (13)	Double-blind, randomized, crossover	Filtered air Diesel exhaust (100 and 200 μg/m^3^)	No changes in prothrombotic endpoints

Mills et al [24] exposed 20 men (mean age 60 years ± 1 year) with prior myocardial infarction to either dilute diesel exhaust (300 μg/m^3 ^from idling Volvo TD45, 4.5 liters, 4 cylinders, 680 rpm diesel engine; median particle size 54 nm) or filtered air for one hour with exercise. The subjects had had a myocardial infarction more than six months prior to enrollment, had been treated with primary angioplasty and stenting, and were receiving standard secondary preventive therapy. In addition to unstable coronary disease, men with angina pectoris, a history of arrhythmia, diabetes mellitus, uncontrolled hypertension, renal failure, and hepatic failure were excluded. Current smokers and men with asthma, significant occupational exposures, or an intercurrent illness were also excluded from the study. This was a double-blind, randomized, crossover study. The two exposures were separated by at least two weeks. The average number of particles was 1.26 × 10^6^/cm^3^. The average concentrations of NO_x_, NO_2_, NO, CO and total hydrocarbon were 4.45 ± 0.02 ppm, 1.01 ± 0.01 ppm, 3.45 ± 0.03 ppm, 2.9 ± 0.1 ppm and 2.8 ± 0.1 ppm respectively. Endpoints included vascular studies (requiring brachial artery cannulation with a 27 gauge needle), plasma tissue plasminogen activator (tPA), plasminogen activator inhibitor-1 (PAI-1), and C-reactive protein (CRP). Heart rate increase with exercise was similar between diesel and filtered air. Myocardial ischemia was detected during exercise in all subjects. Relative to filtered air, there was a greater decrement of ST segment during exposure to diesel exhaust and this was interpreted as an increase in the exercise-induced ischemic burden. Diesel exhaust did not aggravate preexisting vasomotor dysfunction measured as endothelium-dependent (acetylcholine) and endothelium-independent (nitroprusside) vasodilatation at six hours after exposure. Exposure to diesel exhaust did reduce the acute bradykinin-induced release of endothelial tPA. There were no differences in tPA, PAI-1, leukocyte and neutrophil counts, platelet counts, and CRP.

These findings in patients with prior myocardial infarction [24] are in contrast to those observed in healthy subjects published by the same group [29]. These authors exposed 30 healthy men between 20 and 38 years old to diluted diesel exhaust (300 μg/m^3^) or air for one hour using the same exposure protocol. This study of healthy volunteers showed that diesel exhaust attenuated increases in forearm blood flow induced by bradykinin, acetylcholine, and nitroprusside infusion measured two and six hours after exposure. Diesel exhaust also suppressed the bradykinin-induced release in plasma tPA six hours after exposure. Tornqvist et al [30] examined the vascular dysfunction in 15 healthy volunteers 24 hours after exposure to diesel exhaust or filtered air using the same protocol as that of Mills et al. They also found that exposure to diesel exhaust reduced acetylcholine- and bradykinin-induced vasodilatation, but diesel exhaust had no effects on endothelium-independent vasodilatation induced by sodium nitroprusside or verapamil. Unlike the studies by Mills et al [24,29], diesel exhaust did not attenuate bradykinin-induced increase in plasma tPA.

Most recently, Mills et al conducted another study in which 12 patients with stable coronary artery disease and 12 age-matched normal volunteers were exposed to concentrated ambient fine and ultrafine particles or filtered air [31]. The mean particle concentration in the chamber was 190 ± 37 μg/m^3^. They found an approximately three-fold increase in exhaled breath 8-isoprostane after CAPs exposure in both groups. CAPs exposure increased blood flow and plasma tPA in a dose-dependent manner; however, there was no effect on vascular function in either group. Because the ambient particles used in the study were low in elemental carbon, the lack of changes in the markers of vascular functions was attributed to the low combustion-derived particles in CAPs. Of note, patients with coronary artery disease also showed no increase in exhaled breath 8-isoprostane, forearm blood flow and plasma tPA compared to their age matched control [31].

Thus in patients with coronary artery disease, HRV effects and endothelial dysfunction caused by diesel exhaust are diminished or absent compare to those in healthy subjects with no history of cardiovascular disease [24,25,29,30]. Even if the exposure was to particles from low combustion sources, patients with coronary artery disease still show no increased responsiveness compared to their age matched control [31]. These findings were also in contrast to those from some panel studies. For example, endothelial dependent and endothelial independent vascular reactivity reduced only in patients with diabetes [32]. Plasma plasminogen activator inhibitor-1 (PAI1) levels were significantly increased in a group of patients with coronary artery disease when hourly concentrations of PM10 were greater than 100 μg/m3, although the study did no include a control low risk group [33]. PM10 levels were associated positively with increased von Willebrand factor (vWF) only in person with a history of diabetes [34].

### Controlled exposures to ambient pollutant particles in at-risk patient populations

Controlled exposure studies involving at-risk populations that are discussed in this review are summarized in Table 1. Peretz et al exposed 10 healthy subjects and 17 patients with metabolic syndrome to filtered air (FA) and diesel exhaust (DE) at two levels (100 μg/m^3 ^and 200 μg/m^3^) for two hours [35]. Before and after each exposure, they measured the brachial artery diameter (BAd) by B-mode ultrasound and collected blood samples for endothelin-1 (ET-1) and catecholamines. They also assessed endothelium-dependent flow-mediated dilation (FMD) postexposure. Compared with FA, DE dose dependently decreased BAd. DE at 200 μg/m^3 ^increased plasma levels of ET-1. There was no consistent impact of DE on plasma catecholamines or FMD. Patients with metabolic syndrome and healthy subjects had similar DE-induced changes.

Carlsten et al exposed 16 subjects with metabolic syndrome to filtered air and diesel exhaust (DE) at two levels (100 μg/m^3 ^and 200 μg/m^3^) for two hours [36], an exposure protocol the same as that in the study by Peretz et al [35]. They assessed several thrombotic biomarkers, including D-dimer, von Willebrand factor (VWF), and plasmin activator inhibitor-1 (PAI-1) at three, seven, and 22 h after exposure initiation. Except for a small decrease in VWF at 7 h induced by DE at 200 μg/m^3^, there were no changes in other primary endpoints at other time points. A previous study on healthy subjects by Carlsten et al also showed no changes in prothrombotic endpoints after DE exposure [37].

Patients with metabolic syndrome are at risk for developing coronary heart disease. The lack of changes in this at-risk population is in contrast to epidemiological studies that showed increased vWF and hematological markers of inflammation and impaired endothelial function in association with PM exposure [32,38,39].

### Controlled exposures to ambient pollutant particles in patients with obstructive airway diseases

Controlled exposure studies involving patients with obstructive airway diseases that are discussed in this review are summarized in Table 2. Gong et al exposed 12 young healthy and 12 asthmatic volunteers (average FEV_1 _= 89%) to concentrated ambient particulates (CAPS) in the fine (PM_2.5_) size range at an average concentration of 174 μg/m^3^, and to filtered air (FA) with intermittent exercise [26]. Various respiratory and cardiovascular endpoints were measured. PM_2.5 _decreased systolic blood pressure in asthmatics and increased in healthy subjects relative to FA. There were no differences in the other endpoints, including spirometry, blood cell count, cells in induced sputum, blood coagulability, systemic inflammation and HRV, between the healthy and asthmatic volunteers.

**Table 2:** Controlled human exposure studies in patients with obstructive airway diseases.

Study	Subject (number)	Design	Particles	Results
Gong et al [26]	Healthy volunteers (12) Patients with mild asthma (12)	Crossover	Filtered air, Fine CAPS (174 μg/m^3^)	CAPS induced modest and similar increases in parasympathetic stimulation of HRV in both groups

Gong et al [42]	Healthy elderly, age (6) Elderly patients with COPD (13)	Crossover	Filtered air, Fine CAPS (200 μg/m^3^)	Decrease in HRV in healthy elderly but not in COPD patients

Frampton et al [40]	Healthy volunteers (40), Patients with asthma (16)	Crossover	Filtered air Ultrafine carbon particles (10, 25 and 50 μg/m^3^)	In healthy subjects, reduced CD54 and CD18 on monocytes; decreased CD18 and CD49d on granulocytes; decreased CD25 in blood monocytes, basophils, and eosinophils; increased lymphocyte expression of CD25. In asthma, reduced CD11b on monocytes and eosinophils, CD54 on granulocytes, the percentage of CD4+ T cells, basophils, and eosinophils

Piatropaoli et al [41]	Healthy volunteers (40), Patients with asthma (16)	Crossover	Filtered air Ultrafine carbon particles (10, 25 and 50 μg/m^3^)	No changes in airway eNO and total cell count/differentials in induced sputum; Increased percentage macrophages in the sputum of asthmatics; No changes in IL-6 and IL-8 in the sputum

Gong et al [67]	Healthy volunteers (4) Patients with mild asthma (12)	Crossover	Filtered air Coarse CAPS (157 μg/m^3^)	Decrease HR and PNN50 4 hours post-exposure

Stenfors et al [43]	Healthy volunteers (25) Patients with mild asthma (15)	Single-blind randomized crossover	Filtered air Diesel exhaust (PM10, 108 μg/m^3^)	Increased neutrophils, IL-8 and IL-6 in BAL fluid and P-selectin and VCAM-1 in bronchial biopsy, no difference between normal and asthmatics

Gong et al [44]	Healthy volunteers (14) Patients with mild asthma (17)	Crossover	Filtered air Ultrafine CAPS (100 μg/m^3 ^or 145,000/cm^3 ^particle counts	Decreased O2sat, FEV1 and low frequency power; no differences between normal and asthmatics

Frampton et al [40] and Pietropaoli et al [41] exposed 16 asthma patients with normal FEV_1 _to freshly generated elemental ultrafine carbon particles (10 μg/m^3^) or filtered air. Compared to healthy subjects exposed to the same concentration of particles, there were no differences in airway inflammation parameters (including exhaled NO and inflammatory cells in induced sputum). Ultrafine carbon particles exposure reduced expression of adhesion molecules CD54 and CD18 on monocytes and CD18 and CD49d on granulocytes, and increased lymphocyte expression of activation marker CD25 in healthy subjects. Ultrafine carbon particles exposure induced a different leukocyte surface marker response in asthmatics with a reduction in expression of CD11b on monocytes and eosinophils and CD54 on granulocytes.

Gong et al [22] exposed six elderly healthy subjects and 18 volunteers with COPD (mean FEV_1 _= 54%) on separate days to (a) filtered air (FA); (b) 0.4 ppm NO_2_; (c) PM_2.5 _CAPS (~200 μg/m^3^); and (d) CAPS and NO_2 _together. CAPS caused small decreases in maximal mid-expiratory flow and arterial O_2 _saturation and the decrement was greater in healthy than COPD subjects. Similarly, CAPS produced a greater decrease in percentages of columnar epithelial cells in sputum in the healthy subjects. In a similar study by Gong et al [42], CAPS decreased ectopic beats in COPD subjects relative to FA. Heart-rate variability over multi-hour intervals was lower after CAP than after FA in healthy elderly subjects but not in COPD patients.

Stenfors et al exposed 25 non-atopic subjects and 15 patients with mild asthma to two hours of diesel exhaust (DE) (PM10, 108 μg/m^3^) or filtered air [43]. DE increased airway resistance in both groups. During exposures, volunteers alternately exercised on a bicycle ergometer and rested for 15 min periods. Bronchoscopy with bronchial biopsy was performed six hours after ceasing exposure. Healthy subjects, but not asthmatics, had increased neutrophils, IL-8 and IL-6 in BAL fluid and P-selectin and VCAM-1 in bronchial biopsy after DE exposure. Only asthma patients had an increased in IL-10 after DE exposure.

Gong et al [44] exposed adult volunteers (17 healthy, 14 asthmatic) in a controlled environmental chamber to filtered air or concentrated ultrafine particles (UFP) collected in a Los Angeles suburb with substantial motor vehicle pollution. Exposures lasted two hours with intermittent exercise. Inhaled particle counts (mean 145,000/cm^3^, range 39,000-312,000) were typically 7-8 times higher than ambient levels. Mass concentrations (mean 100 μg/m3, range 13-277) were not highly correlated with counts. Relative to filtered air studies, UFP exposures were associated with a 0.5% mean fall in arterial O2 saturation estimated by pulse oximetry (p < 0.01), a 2% mean fall in forced expired volume in one sec (FEV1) the morning after exposure (p < 0.05), and a transient slight decrease in low frequency (sympathetic) power in Holter recordings during quiet rest (p < 0.05). Healthy and asthmatic subjects were not significantly different across most endpoints.

Controlled exposure studies in patients with obstructive airway diseases so far showed that patients with mild asthma appear to have similar or decreased pulmonary and systemic responses to PM exposure compared to healthy volunteers. In addition, while elderly healthy subjects showed changes in ventilatory function and HRV after PM exposure, elderly patients with moderate COPD are relatively unresponsive.

### Factors responsible for the diminished responses in patients with cardiopulmonary diseases

The lack of increased pulmonary and systemic responsiveness to PM in patients with cardiopulmonary diseases during controlled exposures are somewhat surprising since epidemiological studies consistently show that these patients have higher incidence of PM-associated morbidity and mortality [1,3,14,16]. What may account for this lack of increased pulmonary and systemic responsiveness in these susceptible populations during controlled exposure to PM?

The susceptible patients may have impaired baseline values and therefore have decreased response to PM. This may apply to endpoints that have a maximal response, such as blood flow, vasoconstriction and HRV. In the study of Mills et al [24], the baseline forearm blood flow was the same in patients with coronary heart disease compared to matched healthy control. In the study by Routledge et al [25], the HRV parameters were not different between healthy volunteers and patients with coronary artery disease. However, since few investigations include head-to-head comparison between healthy and diseased subjects in the same study, this remains a possibility.

Medications may alter the response to PM exposure. The subjects recruited into the controlled exposure studies are often required to be on fully appropriate medications, a condition that cannot be controlled in epidemiological studies. In the study by Routledge et al. [25], 70% of the patients were on β-blockers, 85% on aspirin and 90% on statins. In the study by Mills et al. [24], all patients were on aspirin, 90% on statins and 75% on a β-blocker. In the studies by Gong et al. [22,42], all COPD patients were on a stable regimen of bronchodilators and/or inhaled steroids. Regularly prescribed β-blockers blunted the association between air pollution and supraventricular arrhythmias in a group of patients with an implanted cardioverter defibrillator [45]. Statins eliminated the association between PM_2.5 _and decreased high frequency power [46]. Not taking statins enhanced the associations between PM_2.5 _and plasma VCAM-1 in patients with type 2 diabetes [39]. The association between personal PM_10 _exposures and blood pressure and brachial arterial flow was only present in diabetic patients not taking vasoactive medications [47]. Both β-blockers and bronchodilators were found to attenuate PM-induced changes in SDNN [48]. The medication effects appear not limited to exposure to PM since the reduction in HRV markers of cardiac vagal control observed in normal volunteers after exposure to SO_2 _was not seen in patients with stable angina [25].

Disease itself may modify the response to PM exposure. Patients with COPD due to α 1-antitrypsin deficiency demonstrated decreased cardiac parasympathetic modulation and changes in these HRV indices positively correlated with FEV_1 _[49]. Thus the baseline autonomic nervous system dysfunction in COPD patients may modify their HRV response to PM exposure [49-51]. Patients with obstructive airway diseases have different patterns of deposition of PM during exposure. Studies examining particle deposition in the lung among patients with obstructive airway diseases showed that lung deposition fraction increased in patients with various obstructive airway diseases [52], but a greater proportion of the particles is deposited in the proximal airways [53,54] resulting in a decrease in effective surface area for particle-lung cell interaction. The decrease in particle-cell interaction may attenuate the biological effects of PM. The altered deposition pattern, however, may only explain part of the attenuated response in COPD patients. One previous study showed that PM-induced increase in SDNN was more prominent in COPD patients whose the baseline FEV_1 _was smaller [48]. These factors may explain the inconsistent associations between PM and various pulmonary and cardiovascular endpoints in a panel study [55].

Subject selection bias may also have contributed to the lack of increased responsiveness observed in controlled exposures of susceptible populations. Unlike epidemiological studies that typically include a large cohort of patients regardless of their risk profiles, all controlled exposure PM studies involving susceptible populations have very strict inclusion and exclusion criteria and thus likely select the most stable patients, in part for safety reasons. These patients tend to have optimal medications and/or have received other disease-modifying interventions, and thus are most "stable".

Patients selected for controlled exposure studies may carry genetic traits that are protective and thus are inherently less responsive than the usual susceptible patients. For example, the PM_2.5_-associated decrease in high frequency power was only detected in subjects without the GSTM1 allele, but not subjects with GSTM1 present [46]. Carriers of C677T methylenetetrahydrofolate reductase (MTHFR) genotypes (CT/TT) had lower SDNN than CC MTHFR subjects [56]. Elderly subjects with the GSTM1 deletion and the HMOX-1 long repeat had decreased SDNN, HF and LF after PM2.5 exposure [57]. Subjects with two hemochromatosis (HFE) gene polymorphisms (C282Y and H63D) appear protected from the effect of particles on cardiac autonomic function compared with wild-type subjects [58].

Some susceptible population may have biologically up-regulated counter-regulatory mechanisms that counter-balance the decreased physiological reserve, or that actually mitigate air pollution exposures. Antioxidant systems in the lung are induced in patients with COPD, and the degree of induction may be sufficient to attenuate oxidative stress induced by PM exposure [59-61].

Selection of study endpoints may also have some effects on the results. Many of the endpoints used in controlled exposure studies are not specific for the disease or condition that the subjects have. For example, HRV is a sensitive measure for autonomic nervous system dysfunction but it may be irrelevant if PM-induced health effects in patients with asthma or COPD are mostly respiratory. The usefulness of a disease-specific endpoint that is related to PM-induced health effects is best illustrated by the study of Mills et al that showed increased ST depression during exercise in patient with coronary artery disease [24] and by the study of Stenfors et al that showed increased IL-10 in asthma patients after exposure to diesel exhaust [43].

### Recommendations for future studies

To date, controlled PM exposure studies in patients with chronic cardiopulmonary diseases have shown some novel results. More controlled exposure studies enrolling these patients are needed to better characterize the effects and mechanisms by which PM causes cardiopulmonary health effects in susceptible populations. Future studies should include endpoints that are more specific to the pathogenesis of the disease and measurements that are most relevant to PM-induced adverse effects in the susceptible population under investigation. Furthermore, all studies should collect and store DNA samples so that genetic polymorphism of each patient in controlled human exposures can be obtained and correlated with the endpoints if necessary. Finally, we may consider controlled exposure using filtered air as the "intervention" rather than the "control" conceptually. To this end, we are reminded of a study that showed decreased leukocyte count and IL-6 in the peripheral blood in a group of expedition members who traveled from a more polluted Japanese metropolitan to the Antarctica that has the particulate number density <1% of that measured in Japan [62]. One may consider a similar study design for controlled exposure in which the susceptible subjects are exposed to ambient levels of PM (with or without gaseous co-pollutants) and filtered air and assess the improvement in the biological endpoints. In this approach, filtered air is considered as "intervention" and the ambient level of PM is regarded as "control" conceptually. This approach could decrease the potential risk of these susceptible subjects from the exposure to high levels of PM and allow us to include patients with more severe disease who are most likely the populations that developed adverse effects with exposure to PM. Although the improvement signal may be small, they may be amplified in these higher risk patients because they have much worse baseline values. The same approach may also be used to study the most vulnerable population, i.e., children. Exposing children to CAPS in controlled exposure studies may generate significant scrutiny and concerns because of the potential effects of increased ambient PM on growth and development of children [63-66].

## Conclusion

Controlled exposure studies remain an integral part of the research in PM-induced health effects. Controlled exposure studies in normal volunteers in the past decades have provided important insights and novel mechanisms for the adverse health effects associated with ambient particle exposure. More controlled exposure studies with novel designs will be needed to better characterize the effects and mechanisms by which PM causes cardiopulmonary health effects in susceptible populations.

## Competing interests

The authors declare that they have no competing interests.

## Authors' contributions

YCH reviewed the references, wrote the first draft and revised the manuscript. AJG reviewed the references and helped revise the manuscript.
